# Safety and Pharmacokinetics of Nirsevimab in Japanese Infants: Primary Analysis of the Open-Label JUBILUS Trial

**DOI:** 10.1093/jpids/piag008

**Published:** 2026-03-24

**Authors:** Masaaki Mori, Susannah Leach, Maria Learoyd, Divya Vijapur, Sam Sadow, Deidre Wilkins, Yoshifusa Abe, Kazushige Ikeda, Hirokazu Kanegane, Zempei Kano, Hiroyuki Moriuchi, Jun Muneuchi, Ryuta Nishikomori, Kaoru Okazaki, Therese Takas, Ayako Sakaguchi, Tonya Villafana

**Affiliations:** Department of Lifelong Immunotherapy, Institute of Science Tokyo, Tokyo, Japan; Division of Rheumatology and Allergology, Department of Internal Medicine, St. Marianna University School of Medicine, Kawasaki, Japan; Vaccines & Immune Therapies, Biopharmaceuticals R&D, AstraZeneca, Gaithersburg, MD, United States; Clinical Pharmacology & Safety Sciences, AstraZeneca, Cambridge, United Kingdom; Global Patient Safety Biopharma, Chief Medical Office, R&D, AstraZeneca, Luton, United Kingdom; Late Vaccine and Immunology, BioPharmaceuticals R&D, AstraZeneca, Gaithersburg, MD, United States; Translational Medicine, Vaccines & Immune Therapies, BioPharmaceuticals R&D, AstraZeneca, Gaithersburg, MD, United States; Children's Medical Center, Showa Medical University Koto Toyosu Hospital, Tokyo, Japan; Division of Neonatology, Department of Pediatrics, Saitama City Hospital, Saitama, Japan; Department of Child Health and Development, Graduate School of Medicine and Dental Sciences, Institute of Science Tokyo Hospital, Tokyo, Japan; Department of General Pediatrics and Interdisciplinary Medicine, Fukuoka Children's Hospital, Fukuoka, Japan; National Research Centre for the Control and Prevention of Infectious Diseases, Nagasaki University Hosptial, Nagasaki, Japan; Department of Pediatrics, Kyushu Hospital, Japan Community Healthcare Organization, Fukuoka, Japan; Department of Pediatrics and Child Health, Kurume University School of Medicine, Kurume, Japan; Department of Neonatology, Tokyo Metropolitan Children's Medical Center, Tokyo, Japan; Vaccines & Immune Therapies, Biopharmaceuticals R&D, AstraZeneca, Gaithersburg, MD, United States; Biopharmaceuticals R&D Japan, AstraZeneca K.K., Tokyo, Japan; Vaccines & Immune Therapies, Biopharmaceuticals R&D, AstraZeneca, Gaithersburg, MD, United States

**Keywords:** infant, monoclonal antibody, nirsevimab, respiratory syncytial virus infections, respiratory tract infection

## Abstract

In Japanese infants aged ≤12 months at risk for severe respiratory syncytial virus-associated lower respiratory tract infections who received two doses of nirsevimab 5–6 months apart, no safety concerns or anti-drug antibodies occurred. Nirsevimab serum concentrations were consistent with those previously demonstrated to be efficacious in healthy preterm and term infants.

## INTRODUCTION

Respiratory syncytial virus (RSV) is one of the most common causes of severe lower respiratory tract infections (LRTIs) in young children.^1^ In 2019 an estimated 1.4 million RSV-associated acute LRTI hospital admissions and 13 300 RSV-associated acute LRTI in-hospital deaths occurred globally in children 0–60 months of age.^2^

Nirsevimab, an extended half-life (~71 days) anti-RSV monoclonal antibody,^3^ is approved by the Japanese Pharmaceuticals and Medical Devices Agency for the prophylaxis of RSV-induced lower respiratory tract disease in all neonates, infants, and children entering their first RSV season, and second season for those at risk of serious RSV infection.^4^ The recently completed real-world phase 3b open-label HARMONIE trial in 8057 infants across France, Germany, and the UK demonstrated 83% efficacy against RSV-associated LRTI hospitalization up to 180 days after randomization during the 2022–2023 RSV season compared with the standard-care group (*P*<.001).^5^ In the open-label, global, phase 3 MUSIC trial in immunocompromised children ≤24 months of age, nirsevimab was well tolerated, with no safety concerns.^6^ Moreover, serum concentrations were similar to those observed in healthy preterm and term infants,^7^ and were therefore supportive of efficacy in this population. The combined clinical and pharmacokinetic (PK) data from these and other trials indicate that the duration of protection afforded by nirsevimab is at least 5–6 months in temperate climates.^5,8^ However, Japan has four distinct climates, ranging from sub-tropical in the South to sub-arctic in the North,^9^ resulting in a prolongation of the RSV season of up to 10 months in some prefectures.^10,11^

In order to account for the variations in RSV seasons in Japan, the JUBILUS trial (NCT06042049), aimed to assess the safety, PK, anti-drug antibody (ADA) levels, and anti-RSV neutralizing antibody (nAb) levels following two doses of nirsevimab given 5–6 months apart in infants at risk for severe RSV-associated LRTI. Here we report the primary analysis over ≥301 days, with a data cutoff of September 20, 2024.

## METHODS

JUBILUS, an open-label single-arm trial, was conducted at nine sites in Japan (Saitama-shi, Koto-ku, Yokohama-shi, Bunkyo-Ku, Fuchu-shi, Fukuoka-shi, Kurume-shi, Kitakyusyu-shi, and Nagasaki-shi). The trial was initiated in July 2023, and completed in July 2025. On Day 1, participants in the first year of life received Dose 1 of nirsevimab as a single, intramuscular (IM) injection; Dose 2 was administered between Day 151 and 180, equivalent to 5–6 months after Dose 1. Dosage was based on body weight: 50 mg if <5 kg; 100 mg if ≥5 kg. All participants were followed until the end of the trial follow-up period (360 days after Dose 2 or last dose administered in the trial; Supplementary Figure 1).

Participants were enrolled if they were ≤12 months of age, were eligible to receive palivizumab in accordance with national or local guidelines, and were at risk for severe RSV disease. The full list of inclusion and exclusion criteria are shown in Supplementary Information 1.

The primary endpoint of this trial was to assess the incidence of treatment-emergent adverse events (AEs), serious AEs (SAEs), AEs of special interest (AESIs; investigator-assessed), and new-onset chronic diseases (NOCDs). The secondary endpoints were to assess the serum PK, ADAs, and anti-RSV nAbs. Exploratory endpoints included the occurrence of medically attended (MA) RSV LRTI, and RSV-associated LRTI hospitalization. Details of determination of AE causality, AESIs, NOCDs, serum sampling, case definition of MA RSV LRTI, statistical analysis, and ethical considerations can be found in Supplementary Information 2.

## RESULTS

Thirty-three participants were enrolled and received Dose 1. The median age (range) at dosing was 1.7 (0.4–11.9) months, with a median gestational age (range) of 35.0 (27–40) weeks (Supplementary Table 1). At Dose 1, the majority of participants were <5 kg (*n* = 25) but by Dose 2, most were ≥5 kg (*n* = 31). The primary reasons for inclusion were preterm birth (*n* = 20), congenital heart disease (*n* = 6), conditions of immunocompromise (*n* = 4), and Down syndrome (*n* = 3); many participants had multiple conditions (Supplementary Table 2). Three participants underwent cardiac surgery with cardiopulmonary bypass during the study, of whom two received a replacement dose (Supplementary Figure 2).

Among participants who received ≥1 dose, all 33 experienced an AE and nine experienced SAEs, seven of which were grade 3 (Supplementary Tables 3 and 4). One SAE (progressing heart valve prolapse) prevented administration of the second dose per the protocol. AE rates in the first few days after each injection were similar and no local or immediate reactions were reported (Supplementary Table 3). From Dose 1 (Day 1) through administration of Dose 2 (Day 151–180), 30/32 participants experienced ≥1 AE and three experienced an SAE (two were grade 3). Following Dose 2 (Day 151–180), through at least Day 301, 31 and six participants experienced an AE and SAE, respectively, of which five SAEs were grade 3. No participants experienced an investigator-assessed AESI, while one was diagnosed with an NOCD of infantile spasms 132 days after receiving Dose 2. None of the AEs, SAEs, AESIs, or NOCDs were considered treatment-related, and no deaths occurred.

**Figure 1:**
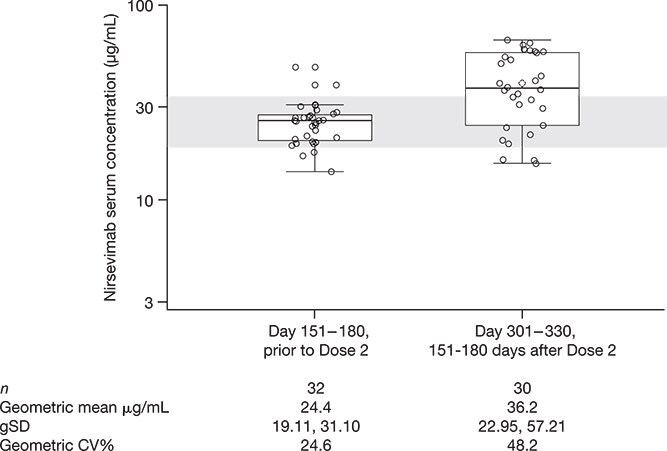
Individual Nirsevimab Serum Concentrations Following Dose 1 and Dose 2 Compared with the Phase 3 MELODY Study. All participants were followed through at least day 301; data cutoff September 20, 2024. Points represent individual participants; boxes represent IQRs; central lines correspond to the medians; whiskers extend to the largest and smallest values no further than 1.5 × IQR. The gray band represents the IQR of healthy infants at day 151 in the MELODY trial.^7^ Three participants underwent cardiac surgery with cardiopulmonary bypass during the study and post-surgery data were not included. Abbreviations: CV, coefficient of variance; gSD, geometric standard deviation; IQR, interquartile range

Nirsevimab serum concentrations were available for 30 participants through Day 301 (Figure 1). Prior to Dose 2 (Day 151–180), the nirsevimab serum geometric mean concentration (GMC) was 24.4 μg/mL (*n* = 32; coefficient of variance 24.6%). The GMC subsequently peaked at the Day 181–210 measurement (30–60 days after Dose 2; *n* = 32; 129.6 μg/mL) and by Day 301 had returned to a similar concentration as that observed at the time of Dose 2 (*n* = 30; 36.2 μg/mL). At the time of data cutoff, all available ADAs samples had a titer of <50 and were therefore reported as ADA negative.

RSV nAb levels increased over time, with the highest levels observed from Day 181–210 (30–60 days after Dose 2; Table 1). Notably, RSV nAb levels were higher on Day 301 (data cutoff) than those observed prior to Dose 2 (Day 151 and 180). nAb levels and nirsevimab serum concentrations were highly correlated (repeated measures correlation estimate: 0.953; 95% CI: 0.931, 0.975).

**Table 1:** RSV Neutralizing Antibody Levels

	**Prior to Dose 1 (Day 1)** **(*n* = 12)**	**Prior to Dose 2 (Day 151–180)** **(*n* = 31)**	**Day 181–Day 210** **(*n* = 15)**	**Day 301–330** **(*n* = 30)**
Geometric mean IU/mL (gSD)	265.5 (3.0)	8127.0 (1.3)	28 552.2 (1.2)	11 241.0 (1.4)
95% CI	132.46, 532.10	7326.11, 9016.24	26 374.21, 30 910.01	9789.06, 12 908.25
Geometric CV%	82.6	18.8	9.5	24.8
Median IU/mL	204.5	7597.0	29 018.0	12 241.0
Range	82–1336	5167–15 944	21 063–33 712	5066–17 577

All participants were followed through at least Day 301; data cutoff September 20, 2024.

Abbreviations: CI, confidence interval; CV, coefficient of variation; gSD, geometric standard deviation; IU, international unit; RSV, respiratory syncytial virus.

There were two incidents of MA RSV LRTI through Day 301. One event occurred on Day 139 and the second on Day 262; neither resulted in hospitalization.

## DISCUSSION

This primary analysis of the open-label JUBILUS trial demonstrated that two doses of nirsevimab given 5–6 months apart to Japanese infants ≤12 months of age at risk for severe RSV-associated LRTI were well tolerated. There were no safety concerns through Day 301, the majority of AEs and SAEs were mild–moderate in severity and none were treatment-related. There were no investigator-assessed AESIs and one NOCD event occurred (not related to treatment).

Overall, safety data were in line with the existing safety profile established through several large clinical and real-world trials in both healthy infants^5,7^ and those at risk of severe disease,^6,12^ including some infants in the MEDLEY study^13^ who received a second dose of nirsevimab ahead of their second RSV season (200 mg regardless of weight). Among the participants who experienced respiratory-related SAEs in JUBILUS (bronchitis, viral bronchitis, and viral sepsis), the nature, timing, and clinical course of these SAEs was consistent with the participants’ underlying conditions and further supports the safety profile of nirsevimab.

Nirsevimab serum concentrations and RSV nAb levels increased as expected, with the highest levels observed 1 month after Dose 2. Notably, nirsevimab serum concentrations at Day 151–180 prior to Dose 2 and at Day 301–330, 151–180 days after Dose 2 were consistent with those observed in healthy term and preterm infants in the phase 3 MELODY trial where efficacy was established (mean ± standard deviation: 19.6 ± 7.7 μg/mL in participants <5 kg and 31.2 ± 13.7 μg/mL in participants ≥5 kg; Figure 1)^7^ and is therefore supportive of efficacy in this population. Nirsevimab serum concentrations were also similar to those in the phase 3 MUSIC trial of nirsevimab in immunocompromised children ≤24 months of age.^6^ nAb levels were highly correlated with nirsevimab serum concentrations.

All ADA samples through to Day 301 were negative, which is comparable with the low levels of ADAs observed in the MELODY (6.1%)^7^ and MUSIC (11%)^6^ trials.

Two participants (6.1%) experienced an MA RSV LRTI, neither of which resulted in hospitalization. As both participants had a number of other respiratory events and infections during the trial, aside from the confirmed RSV infection, MA RSV LRTIs were not unexpected for these individuals. Notably, this was an exploratory endpoint, and given the low patient number, no conclusions can be drawn from these data.

The main limitation of this study was the low number of participants; however, this was appropriate for a study of this nature and in this population. Additionally, as many participants were born preterm, some blood samples were of insufficient volume for testing, leading to missing data.

In conclusion, this primary analysis shows that for Japanese infants ≤12 months of age at risk for severe RSV-associated LRTI, the safety profile of two doses of nirsevimab given 5–6 months apart was consistent with that published previously for single doses in healthy participants. No new safety signals were detected, no ADAs were observed, and the incidence of MA RSV LRTI was low through Day 301. Nirsevimab serum concentrations were supportive of protection in this population.

## Supplementary Material

piag008_Supplemental_Files

## Data sharing statement

Data underlying the findings described in this manuscript may be obtained in accordance with AstraZeneca’s data sharing policy described at https://astrazenecagrouptrials.pharmacm.com/ST/Submission/Disclosure. Data for studies directly listed on Vivli can be requested through Vivli at www.vivli.org. Data for studies not listed on Vivli could be requested through Vivli at https://vivli.org/members/enquiries-about-studies-not-listed-on-the-vivli-platform/. The AstraZeneca Vivli member page is also available outlining further details: https://vivli.org/ourmember/astrazeneca/.
