# Supplementary material for: Safety and Pharmacokinetics of Nirsevimab in Japanese Infants: Primary Analysis of the Open-Label JUBILUS Trial
Source: J Pediatric Infect Dis Soc. 2026 Mar 24;15(3):piag008. doi: 10.1093/jpids/piag008 (PMC13025070; doi:10.1093/jpids/piag008)
Supplement: piag008_Supplemental_Files [file piag008_supplemental_files.zip › Mori_JUBILUS manuscript supplement_revised_piag008.docx]

**Safety and Pharmacokinetics of Nirsevimab in Japanese Infants: Primary Analysis of the Open Label JUBILUS Trial**

Masaaki Mori^1,2^; Susannah Leach^3^; Maria Learoyd^4^; Divya Vijapur^5^; Sam Sadow^6^; Deidre Wilkins^7^; Yoshifusa Abe^8^; Kazushige Ikeda^9^; Hirokazu Kanegane^10^; Zempei Kano^11^; Hiroyuki Moriuchi^12^; Jun Muneuchi^13^; Ryuta Nishikomori^14^; Kaoru Okazaki^15^; Therese Takas^3^; Ayako Sakaguchi^16^; Tonya Villafana^3^

# SUPPLEMENTARY INFORMATION

## Supplementary Information 1. Inclusion and Exclusion Criteria

### Inclusion Criteria

Participants were eligible to be included in the trial only if all of the following criteria applied:

1. Written informed consent and any locally required authorization obtained from the participant’s parent(s)/legally authorized representative(s) before performing any protocol-related procedures, including screening evaluations

2. Japanese infants of ≤12 months of age eligible to receive palivizumab in accordance with national or local guidelines and those who must meet at least one of the following conditions at the time of informed consent.

(a) Immunodeficiency

i) Diagnosed with combined immunodeficiency (severe combined immunodeficiency, X-linked hyper-immunoglobulin M [IgM] syndrome, etc.); antibody deficiency (X-linked agammaglobulinemia, common variable immunodeficiency, non-X-linked hyper-IgM syndromes, etc.); or other immunodeficiencies (Wiskott-Aldrich syndrome, DiGeorge syndrome, etc.), or

ii) Diagnosed with human immunodeficiency virus infection, or

iii) History of organ or bone marrow transplantation, or

iv) Participant is receiving immunosuppressive chemotherapy, or v) Participant is receiving systemic high-dose corticosteroid therapy (prednisolone equivalents ≥0.5 mg/kg every other day, other than inhaler or topical use), or

vi) Participant is receiving other immunosuppressive therapy (eg, azathioprine, methotrexate, mizoribine, mycophenolate mofetil, cyclophosphamide, cyclosporine, tacrolimus, cytokine inhibitors, etc.)

(b) Chronic lung disease (CLD)

i) Diagnosed with CLD of prematurity requiring medical intervention/management (ie, supplemental oxygen, bronchodilators, or diuretics) within 6 months prior to screening

(c) Congenital heart disease (CHD)

i) Diagnosed with hemodynamically significant CHD (must be unoperated or partially corrected CHD)

Note: Infants with hemodynamically significant acyanotic cardiac lesions must have pulmonary hypertension (≥40 mmHg measured pressure in the pulmonary artery) or the need for daily medication to manage CHD

(d) Down syndrome

(e) Born preterm ≤28 weeks gestational age (GA) and ≤12 months of age, or born preterm >28 weeks and ≤35 weeks GA and ≤6 months of age

3. The participant’s parent(s)/legally authorized representative(s) can understand and comply with the requirements of the protocol including follow-up visits as judged by the investigator.

4. The participant is available to complete the follow-up period for approximately 19 months, which will be approximately 1 year after receipt of Dose 2 of nirsevimab.

### Exclusion Criteria

Participants were excluded from the study if any of the following criteria applied:

1. Requirement for mechanical ventilation, extracorporeal membrane oxygenation, continuous positive airway pressure, or other mechanical respiratory or cardiac support at the time of enrollment
2. A current, active respiratory syncytial virus (RSV) infection at the time of screening and investigational product administration
3. Any fever (≥100.4°F [≥38.0°C], regardless of route) or acute illness at the time of investigational product administration
4. Any serious concurrent medical condition (except those resulting in an immune deficiency condition), including:
   - 1. Known renal impairment
     2. Known hepatic dysfunction including known or suspected active or chronic hepatitis infection
     3. Any seizure disorder or evolving or unstable neurological condition
5. Anticipated cardiac surgery within 5–6 months after enrollment
6. Prior history of a suspected or actual acute life-threatening event
7. Receipt or intended use of palivizumab in the current enrollment season
8. Any known allergy or history of allergic reaction to any component of nirsevimab
9. Any known allergy or history of allergic reaction to immunoglobulin products, blood products, or other foreign proteins
10. Concurrent enrollment in another interventional study, or prior receipt of any investigational agent
11. Anticipated survival of less than 1 year at the time of informed consent
12. Any condition that, in the opinion of the investigator, would interfere with the evaluation of the investigational product or interpretation of study results
13. Infants of employees of the Sponsor, clinical study site, or any other individuals involved with the conduct of the study, or immediate family members of such individuals

## Supplementary Information 2. Additional methodological information

### Treatment-related adverse events

Adverse events (AEs) were coded according to the Medical Dictionary for Regulatory Activities version 27.1. AE relatedness to treatment was determined by the investigator. Causality of “related” was made if there was evidence for a “reasonable possibility” of a causal relationship for the individual case and was determined on the basis that the available data included enough information to make an informed judgment. With no available facts or arguments to suggest a causal relationship, the event(s) was assessed as ‘not related’.

### Adverse events of special interest

Adverse events of special interest (AESIs) were investigator-assessed and included immediate (type 1) hypersensitivity reactions, such as anaphylaxis, immune complex disease (eg, vasculitis, endocarditis, neuritis, glomerulonephritis), and thrombocytopenia.

### New-onset chronic disease

New-onset chronic disease (NOCD) was defined as a newly diagnosed medical condition of a chronic, ongoing nature that began after treatment was administered and was assessed by the investigator as being medically significant, e.g. diabetes, autoimmune disease (eg, lupus, rheumatoid arthritis), and neurological disease (eg, epilepsy).

### Serum sampling, pharmacokinetics and antidrug antibodies

For this analysis, serum samples were collected before first dose on Day 1, then prior to the second dose (Day 151–180), followed by between Day 181 and 210 (30–60 days after Dose 2; ±7 days), then between Day 301 and 330 (150–180 days after Dose 2; ±15 days); and whenever participants required hospitalization for respiratory illnesses (**Supplementary Figure 1**). Nirsevimab serum concentrations were measured using a validated enzyme-linked immunosorbent assay (MedImmune, Gaithersburg, MD, USA). Antidrug antibodies (ADA) assessments were performed using a validated electrochemiluminescent assay,[1] for which serum samples were obtained before dosing and after dose administration on days 31, 151 and 361. A dilution of 1:50 was the minimum serum dilution that maximized the ability to detect ADA while minimizing interference from serum matrix components, therefore, ADA titers ≥50 were considered positive.

### Case definition of MA RSV LRTI

The case definition of medically attended (MA) RSV lower respiratory tract infection (LRTI) required confirmation of RSV infection by polymerase chain reaction along with ≥1 finding from the lower respiratory tract and ≥1 clinical sign of severe disease, defined as follows[2]:

1. The presence of signs indicative of lower respiratory tract disease
   - Rhonchi
   - Rales
   - Crackles
   - Wheeze
2. The presence of ≥1 clinical sign of severe disease:
   - Increased respiratory rate (≥60 breaths/min for <2-month-old; ≥50 breaths/min for 2–6-month-old; ≥40 breaths/min for 6–24-month-old)
   - Hypoxemia in room air (O_2_ <95% at ≤1800 m; O_2_ <92% at >1800 m)
   - New-onset apnea
   - Retractions
   - Nasal flaring
   - Grunting
   - Acute hypoxic or ventilatory failure
   - Dehydration due to respiratory distress requiring IV hydration

### Statistical Analysis

Thirty-three participants were enrolled; with an estimated 7% participant drop-out rate between Doses 1 and 2 (based on 5% drop-out from Season 1 in the MEDLEY trial[3]), this allowed for approximately 30 participants to be exposed to two intramuscular (IM) doses, with 79% probability of observing ≥1 AEs if the true event rate was 5%. Thus, 33 participants were considered sufficient to provide adequate sample size to evaluate safety and to describe nirsevimab serum concentrations, ADA and anti-RSV neutralizing antibody (nAb) levels following Dose 2.

The primary analysis was conducted when all enrolled participants had been followed through at least Day 301 (approximately 150 days after Dose 2). Safety was evaluated among a) all participants that received ≥1 dose and b) all participants that received both doses. Pharmacokinetic (PK) analyses were conducted among all participants who received ≥1 dose and had ≥1 quantifiable serum PK observation (lower limit of quantification 0.5 mg/mL). ADA and anti-RSV nAb analyses were performed on participants who received both doses and who had ≥1 corresponding quantifiable measurement after Dose 2. In the event of cardiopulmonary bypass, post-surgery data were not included in the analysis.

### Ethics

This trial was performed in accordance with Good Clinical Practice, including the Declaration of Helsinki. The protocol and informed consent forms were approved by the appropriate institutional review boards or independent ethics committees. Written informed consent was obtained for each participant before screening.

Supplementary Table 1. Demographics and Baseline Characteristics

| Characteristic | Total  (*N* = 33) |
| --- | --- |
| Age at dosing, |  |
| Mean months (SD) | 2.3 (2.4) |
| Median months (range) | 1.7 (0.4–11.9) |
| Sex, n (%) |  |
| Male | 18 (54.5) |
| Female | 15 (45.5) |
| Weight group for Dose 1, Day 1, n (%) |  |
| <5 kg | 25 (75.8) |
| ≥5 kg | 8 (24.2) |
| Weight group for Dose 2, n (%)^a^ |  |
| <5 kg | 1 (3.1) |
| ≥5 kg | 31 (96.9) |
| Gestational age, median weeks (range) | 35.0 (27‒40) |
| Primary reason for inclusion,^b^ n (%) |  |
| Preterm birth | 20 (60.6) |
| Congenital heart disease | 6 (18.2) |
| Conditions of immunocompromise | 4 (12.1) |
| Down syndrome | 3 (9.1) |

^a^For Dose 2, *n* = 32.
^b^Diagnosed with combined immunodeficiency, antibody deficiency, or other immunodeficiency (*n* = 4); history of organ or bone marrow transplantation (*n* = 1); participant is receiving other immunosuppressive therapy (*n* = 1). Participants may have had >1 disorder/condition. While infants with CLD of prematurity were eligible for JUBILUS, no participants with CLD were enrolled.
Abbreviations: CLD, chronic lung disease; SD, standard deviation.

## Supplementary Table 2. Medical History Relevant to Inclusion Criteria

| System Organ Class, *n* (%)  Higher level group  Preferred term | Total  (*N* = 33) |
| --- | --- |
| Any medical history | 33 (100) |
| Congenital, familial and genetic disorders^a^ | 21 (63.6) |
| Cardiac and vascular disorders congenital |  |
| Patent ductus arteriosus | 6 (18.2) |
| Atrial septal defect | 4 (12.1) |
| Ventricular septal defect | 4 (12.1) |
| Atrioventricular septal defect | 1 (3.0) |
| Bicuspid pulmonary valve | 1 (3.0) |
| Congenital arterial malformation | 1 (3.0) |
| Congenital cardiovascular anomaly | 1 (3.0) |
| Congenital pulmonary valve disorder | 1 (3.0) |
| Double outlet right ventricle | 1 (3.0) |
| Fallot's tetralogy | 1 (3.0) |
| Persistent left superior vena cava | 1 (3.0) |
| Pulmonary artery stenosis congenital | 1 (3.0) |
| Chromosomal abnormalities, gene alterations and gene variants^a^ | |
| Trisomy 21 | 3 (9.1) |
| Chromosomal deletion | 1 (3.0) |
| Smith-Magenis syndrome | 1 (3.0) |
| Immune system disorders congenital^a^ |  |
| Bruton's agammaglobulinemia | 2 (6.1) |
| Combined immunodeficiency | 1 (3.0) |
| Hyper IgM syndrome | 1 (3.0) |
| Immune system disorders^a^ |  |
| Graft versus host disease in skin | 1 (3.0) |
| Hypogammaglobulinemia | 1 (3.0) |
| Pre-engraftment immune reaction | 1 (3.0) |

^a^Participants may have had >1 disorder/condition.

**Supplementary Table 3.** Overall Summary of Adverse Events

|  | As Treated Set 1 (Received ≥1 dose) (*n* = 33) | As Treated Set 2 (Received 2 doses) (*n* = 32) | | |
| --- | --- | --- | --- | --- |
| Participants with^a^ | From Dose 1 (Day 1) through Day 301 (data cutoff) (*n* = 33) | From Dose 1 (Day 1) through administration of Dose 2 (Day 151–180) | From Dose 2 (Day 151– 180) through at least Day 301 (data cutoff) | From Dose 1 (Day 1) through at least Day 301 (data cutoff) |
| ≥1 AE, n (%) | 33 (100) | 30 (93.8) | 31 (96.9) | 32 (100) |
| Within 7 days of Dose 1^b^ | 2 (6.1) | 2 (6.3) | ̶ | 2 (6.3) |
| 8‒14 days from Dose 1 | 3 (9.1) | 3 (9.4) | ̶ | 3 (9.4) |
| >14 days from Dose 1 | 33 (100) | 30 (93.8) | ̶ | 32 (100) |
| Within 7 days of Dose 2^c^ | ̶ | ̶ | 4 (12.5) | 4 (12.5) |
| 8‒14 days from Dose 2 | ̶ | ̶ | 1 (3.1) | 1 (3.1) |
| >14 days from Dose 2 | ̶ | ̶ | 31 (96.9) | 31 (96.9) |
| ≥1 treatment-related AE | 0 | 0 | 0 | 0 |
| ≥1 AE of grade 3^d^ severity or higher | 9 (27.3) | 2 (6.3) | 6 (18.8) | 8 (25.0) |
| ≥1 treatment-related AE of grade ≥3^d^ | 0 | 0 | 0 | 0 |
| ≥1 SAE,^e^ n (%) | 9 (27.3) | 3 (9.4) | 6 (18.8) | 8 (25.0) |
| Within 7 days of Dose 1 | 0 | 0 | ̶ | 0 |
| 8‒14 days from Dose 1 | 1 (3.0) | 1 (3.1) | ̶ | 1 (3.1) |
| >14 days from Dose 1 | 8 (24.2) | 7 (21.9) | ̶ | 7 (21.9) |
| Within 7 days of Dose 2 | ̶ | ̶ | 0 | 0 |
| 8‒14 days from Dose 2 | ̶ | ̶ | 1 (3.1) | 1 (3.1) |
| >14 days from Dose 2 | ̶ | ̶ | 6 (18.8) | 6 (18.8) |
| ≥1 treatment-related SAE^e^ | 0 | 0 | 0 | 0 |
| ≥1 SAE^e^ of grade ≥3^d^ | 7 (21.2) | 2 (6.3) | 5 (15.6) | 7 (21.9) |
| ≥1 treatment-related SAE^e^ of grade ≥3^d^ | 0 | 0 | 0 | 0 |
| Any AE with outcome death, n (%) | 0 | 0 | 0 | 0 |
| AE leading to discontinuation, n (%) | 1 (3.0) | 0 | 0 | 0 |
| SAE^e^ leading to discontinuation, n (%) | 1 (3.0) | 0 | 0 | 0 |
| ≥1 AESI^f^ based on investigator assessment | 0 | 0 | 0 | 0 |
|  |  |  |  |  |
| ≥1 NOCD | 0 | 0 | 1 (3.1)^g^ | 1 (3.1)^g^ |
| ≥1 treatment-related NOCD | 0 | 0 | 0 | 0 |

All participants were followed through at least Day 301; data cutoff September 20, 2024. Severity of AEs was graded according to the National Cancer Center Institute Common Terminology Criteria for Adverse Events.
^a^Participants with multiple events in the same category were counted once in that category; participants with events in >1 category were counted once in each of those categories.
^b^One participant had an event of otitis externa and one had diarrhea within 7 days of Dose 1; neither were treatment related.
^c^One participant had an event of bronchitis, one had chilblains, one had rhinitis, and one had both an upper respiratory tract infection and asthma within 7 days of Dose 2; none were treatment related.
^d^An AE of grade 1 denotes a mild event, grade 2 a moderate event, grade 3 a severe event, grade 4 a life-threatening event, and threatening, required inpatient hospitalization, prolongation of existing hospitalization, persistent or significant disability/incapacity, important grade 5 a fatal event.
^e^SAE criteria: death, life- medical event, congenital anomaly/birth defect.
^f^Includes type I hypersensitivity (including anaphylaxis), immune complex disease, and thrombocytopenia.
^g^One case of infantile spasm occurred.
Abbreviations: AE, adverse event; AESI, adverse event of special interest; NOCD, new-onset chronic disease; SAE, serious adverse event.

**Supplementary Table 4.** All serious adverse events by participant and severity (As Treated Set 2)

|  | **Anonymized participant number** | **SAE** | **Study Day at start of event, relative to previous dose** | **Maximum severity^a^** | **Outcome** |
| --- | --- | --- | --- | --- | --- |
| **From Dose 1 (Day 1) through administration of Dose 2 (Day 151–180)** | 1 | Bronchitis viral^b^ | 153 | 2 | Recovered |
|  | 2 | Viral sepsis^b^ | 94 | 3 | Recovered |
|  |  | HCoV-OC43 infection | 134 | 2 | Recovered |
|  | 3 | Apnea | 8 | 3 | Recovered |
| **From Dose 2 (Day 151– 180) through at least Day 301 (data cutoff)** | 4 | Thermal burn | 12 | 3 | Recovered |
|  |  | Infantile spasms | 132 | 3 | Not recovered |
|  | 5 | Metapneumovirus pneumonia | 110 | 3 | Recovered |
|  | 6 | Bronchitis^b^ | 108 | 3 | Recovered |
|  | 7 | Pleural effusion | 204 | 2 | Not recovered |
|  | 8 | Pneumonia bacterial | 101 | 3 | Recovered |
|  | 9 | Asthma | 122 | 3 | Not recovered |

^a^by CTCAE grade

^b^These respiratory infections were transient and recovered within 6 to 17 days from onset with the standard of care.

CTCAE, Common Terminology Criteria for Adverse Events; HCoV-OC43, human coronavirus OC43; SAE, severe adverse event.

**Supplementary figure 1:** Trial design diagram


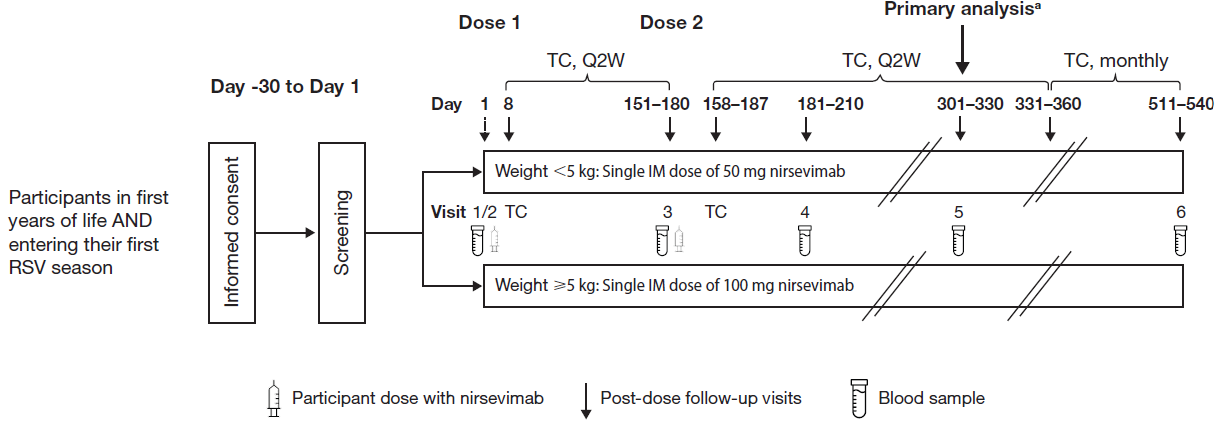


^a^All enrolled participants were followed through at least Day 301; data cutoff September 20, 2024.
Abbreviations: IM, intramuscular; Q2Q, once every two weeks; RSV, respiratory syncytial virus; TC, telephone contact.

**Supplementary figure 2.** Participation Disposition (CONSORT diagram)

**
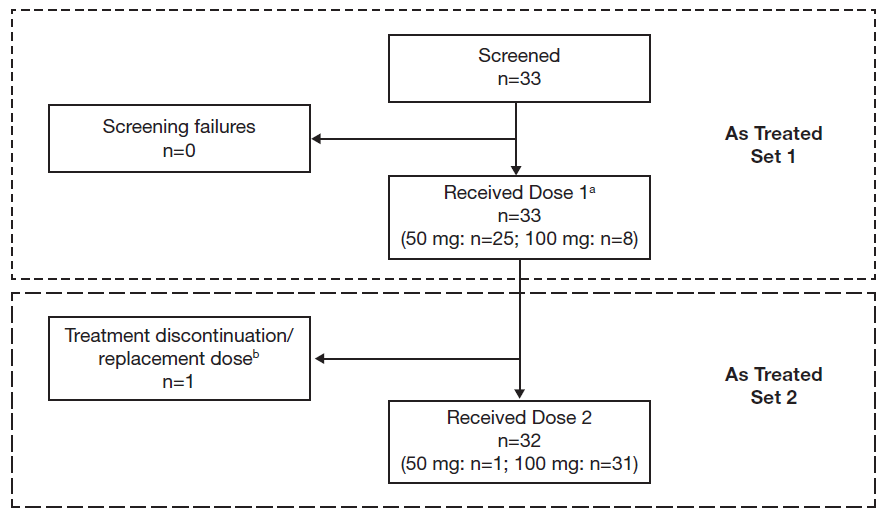
**

All participants were followed for safety. Two participants received a replacement dose following bypass surgery, 1 of whom discontinued.
^a^One participant received a replacement for Dose 1 following bypass surgery.
^b^One participant had Dose 2 delayed then abandoned due to progressing valve prolapse.

# References

1. Domachowske JB, Khan AA, Esser MT, et al. Safety, tolerability and pharmacokinetics of MEDI8897, an extended half-life single-dose respiratory syncytial virus prefusion F-targeting monoclonal antibody administered as a single dose to healthy preterm infants. *Pediatr Infect Dis J* **2018**;37(9):886–92.

2. Villafana T, Falloon J, Griffin MP, Zhu Q, Esser MT. Passive and active immunization against respiratory syncytial virus for the young and old. *Expert Rev Vaccines* **2017**;16(7):1-13.

3. Domachowske J, Madhi SA, Simões EAF, et al. Safety of nirsevimab for RSV in infants with heart or lung disease or prematurity. *N Engl J Med* **2022**;386(9):892-94.
